# Metagenomic next-generation sequencing enhances diagnosis of fungal infections in kidney transplant recipients: a retrospective study

**DOI:** 10.3389/fcimb.2025.1667475

**Published:** 2026-01-26

**Authors:** Qin Wang, Handong Ding, Zongyao Hao, Guiyi Liao

**Affiliations:** 1 Department of Pharmacy, the First Affiliated Hospital of Anhui Medical University, Hefei, Anhui, China; 2 The Grade 3 Pharmaceutical Chemistry Laboratory of State Administration of Traditional Chinese Medicine, Hefei, Anhui, China; 3 Department of Urology, the First Affiliated Hospital of Anhui Medical University, Hefei, China; 4 Institute of Urology, Anhui Medical University, Hefei, China; 5 Anhui Province Key Laboratory of Genitourinary Diseases, Anhui Medical University, Hefei, Anhui, China

**Keywords:** metagenomic next-generation sequencing (mNGS), fungal infections, kidney transplantation, donor-derived infection, antibiotic treatment

## Abstract

**Background:**

Although fungal infections are relatively rare, they have low detection rates and high mortality rates. The value of metagenomic next-generation sequencing (mNGS) in kidney transplant patients with fungal infections remains insufficiently explored, especially regarding diagnosis and antimicrobial stewardship.

**Methods:**

From September 2021 to August 2023, 234 kidney transplant patients were enrolled, with detailed data collected on 66 patients suspected of fungal infections. The pathogen detection performance of mNGS and conventional microbiological tests (CMTs) was compared. The impacts of mNGS and CMTs on treatment adjustment were also assessed. Finally, the value of mNGS in detecting donor-derived infections was investigated.

**Results:**

Among 66 patients, 21 fungal species were identified: 18 species detected by mNGS and 10 by CMTs. The overall positive rate of mNGS was significantly higher than culture (90.67% vs. 26.67%), especially for multiple fungal infections (9vs0). mNGS identified more *Candida* (26vs12), *Pneumocystis jirovecii* (14vs0), *Aspergillus* (10vs4), *Mucor* (6vs2) organisms compared with CMTs. Donor-derived fungi were identified in 11 (6.7%) patients, including 10 cases of *Candida* spp. and 1 case of *Mucor* spp. Anti-infection therapies were adjusted in 28 (24.4%) cases based on mNGS.

**Conclusion:**

The mNGS technique showed distinct advantages in detecting fungal infections in kidney transplant patients, facilitating informed anti-infection strategies and enhanced graft protection. Moreover, it provides effective identification of fungal infections originating from donor sources.

## Introduction

Kidney transplant recipients face substantially elevated risks of life-threatening fungal infections due to chronic immunosuppression. Although invasive fungal infections represent less than 5% of post-transplant complications (Anastasopoulos et al., 2015; Sommerer et al., 2022), they carry disproportionately high mortality rates (Pappas et al., 2010; Singh and Husain, 2013; Silva et al., 2018). Donor-derived infection (DDI), while uncommon, results in significant morbidity and mortality, with approximately one-third of recipients experiencing graft loss or death - rates that are notably elevated in cases involving fungal diseases (Kaul et al., 2021). The reported incidence of DDI is approximately 0.2% among all deceased donor organ transplantations (Ison and Grossi, 2013), with fungal infections accounting for approximately 15.5% of DDI (Shingde et al., 2018). Timely diagnosis remains a major challenge, as conventional culture methods are slow and insensitive, and non-culture-based assays have significant limitations (Anesi and Baddley, 2016).

Metagenomic next generation sequencing (mNGS) addresses the limitations of current diagnosis and has been increasingly applied to pathogen detection in clinical practice. Prior antibiotic exposure does not affect it, enabling faster and more accurate identification. Some studies have demonstrated that mNGS can improve the sensitivity of fungal infection (Zhao et al., 2022; Shi et al., 2023). In contrast, identification of filamentous molds, such as *Aspergillus*, by mNGS remains challenging due to difficulties in extracting DNA from thicker polysaccharide cell walls and the relatively low fungal load in bronchoalveolar lavage fluid (BALF) (Clarke et al., 2018; Han et al., 2019).

However, few studies have investigated the utility of mNGS for diagnosing fungal infections in renal transplant patients. Furthermore, its role in detecting and characterizing donor-derived fungal infections remains largely uninvestigated. In this study, we used mNGS to identify pathogens from multiple sample types in patients with suspected fungal infections, evaluated the types and prevalence of detected pathogens, compared the results with those obtained by conventional methods, and evaluated the clinical impact on antimicrobial therapy management. Crucially, this study provides novel insights into the potential of mNGS for uncovering donor-derived fungal infections, an area where current diagnostic approaches are insufficient.

## Materials and methods

### Patients and study design

This retrospective study was conducted among kidney transplant recipients at the First Affiliated Hospital of Anhui Medical University between September 2021 and August 2023. Given that deceased donors are typically hospitalized in intensive care unit (ICU) for prolonged periods and face elevated infection risks, all organ preservation fluids from donors underwent both conventionally cultured and next-generation sequencing. Of 395 samples collected from 234 transplant recipients initially screened, 75 samples from 66 patients met the inclusion criteria for final analysis. Patients were included if they satisfied the following criteria: (1) availability of complete smear, culture, and mNGS results; (2) mNGS or CMTs identified the fungus. Exclusion criteria comprised: (1) unpaired mNGS and CMTs testing (i.e., not conducted simultaneously or on the same day); (2) incomplete medical records. This study received approval from the Research Ethics Committee of the First Affiliated Hospital of Anhui Medical University. Individual consent for this retrospective analysis was waived in accordance with institutional guidelines.

Clinical specimens were collected following standardized protocols. Each specimen was divided into two parts: one for mNGS analysis and another for traditional culture. Patients with suspected fungal infections additionally underwent serum (1,3)-β-D Glucan (BDG) and serum galactomannan testing. The standards and methods were implemented following the routine microbial culture process established by the Clinical Laboratory of the First Affiliated Hospital of Anhui Medical University.

### Microbiologic methods

Using conventional microbiologic methods, samples (blood, BALF, organ preservation solutions, drainage fluid, sputum) were processed via laboratory staining and cultures. Serum BDG was detected according to the manufacturer’s instructions. Both BALF and serum galactomannan detection were performed using a double-sandwich ELISA, strictly following manufacturer’s protocols.

### Clinical data collection

Clinical data comprising patient demographics, laboratory test results, diagnosis, treatment, and clinical outcomes, were collected from the electronic medical records of the First Affiliated Hospital of Anhui Medical University using a standardized data collection form. Information regarding initial antibiotic and later adjustment based on mNGS results was also collected.

### Metagenomic next-generation sequencing and analysis

A total of 75 clinical samples were collected, comprising bronchoalveolar lavage fluid (BALF, n=23), drainage fluid (n=23), blood (n=13), organ preservation fluid (n=15), and sputum (n=1). Metagenomic sequencing was performed using either DNA-only or combined DNA and RNA protocols.

The samples were sealed aseptically and stored at -20 °C or transported on dry ice to Hugobiotech Co., Ltd., (Beijing, China) to perform mNGS detection immediately. The DNA was extracted and purified according to the instructions of QIAamp DNA Micro Kit (QIAGEN, Hilden, Germany). DNA concentration and quality were checked through Qubit 3.0 Fluoremeter (Invitrogen, Q33216) and agarose gel electrophoresis (Major Science, UVC1-1100).

DNA library construction was performed according to the Qiagen library construction kit (QIAseq Ultralow Input Library Kit) operating instructions. Library quality control was performed by Qubit 3.0 Fluoremeter (Invitrogen, Q33216) and Agilent 2100 Bioanalyzer (Agilent Technologies, Palo Alto, USA). Qualified DNA libraries with different barcode tags were pooled and then sequenced using the Illumina Nextseq 550 sequencing platform (Illumina, San Diego, USA) and a SE75bp sequencing strategy.

After obtaining the sequencing data, high quality data was generated by filtering out connectors, low quality, low complexity, and shorter sequences. Next human-derived sequences matching to the human reference database (hg38) were removed by using SNAP software. The remaining data were then aligned to the microbial genome database using Burrow-Wheeler Alignment. This database contains a large collection of microbial genomes from NCBI containing more than 30,000 microorganisms, including 17,748 species of bacteria, 11,058 species of viruses, 1,134 species of fungi, and 308 species of parasites.

### Criteria for a positive mNGS result

1. For bacteria other than TB, fungi other than Cryptococcus and parasites: sequencing coverage in the top 10 of all pathogens detected and not detected in the negative control (NTC); or sample/NTC with an RPM (reads per million mapped reads) ratio greater than 10.
2. For viruses, tuberculosis and cryptococci: at least 1 specific sequence was detected and not detected in the NTC; or the RPM ratio of sample/NTC was greater than 5.

### Statistical analysis

Normality of continuous variables was assessed using the Shapiro-Wilk test. Normally distributed data are presented as mean ± SD, and non-normally distributed data as median (IQR). Categorical variables were compared using the chi-square test or Fisher’s exact test where appropriate. Paired binary comparisons (e.g., mNGS vs. CMTs) were analyzed using the McNemar test. A two-tailed p-value of <0.05 was considered statistically significant.

## Results

### Sample and patient characteristics

**Figure 1:**
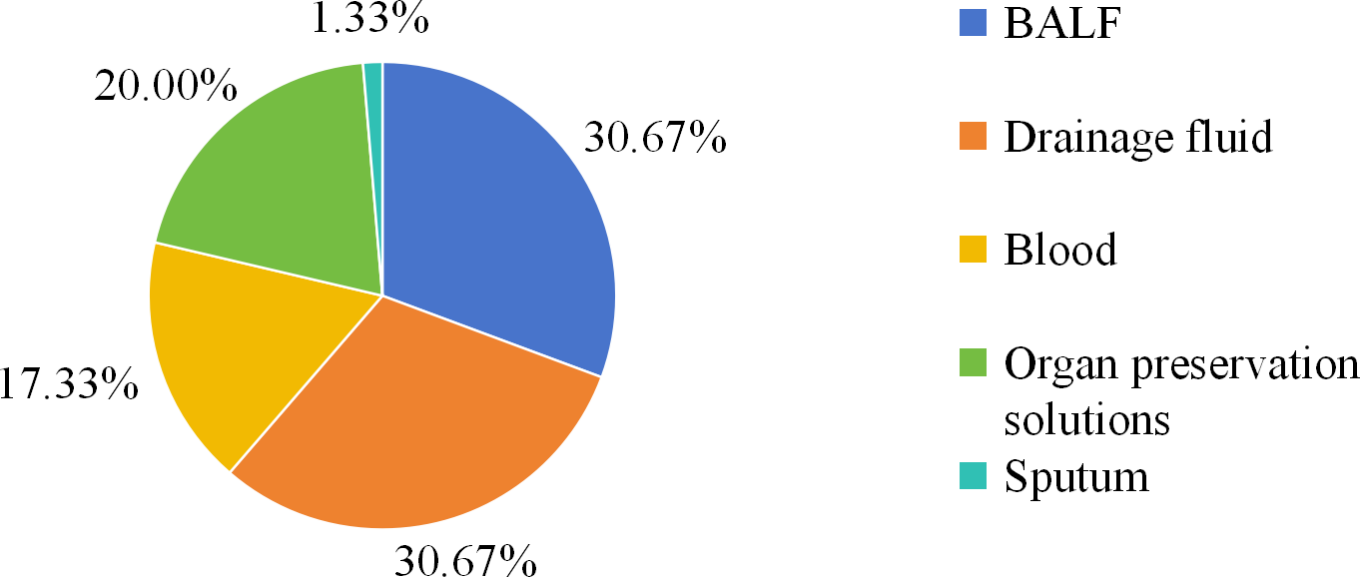
Distribution of sample types; BALF, bronchoalveolar lavage fluid.

Between September 2021 and August 2023, 75 samples from 66 patients with suspected fungal infections were analyzed. All patients underwent both mNGS and CMTs. The mean patient age was 43.7 ± 9.6 years, with 39 (59.09%) males. The most prevalent comorbidity was anemia (53.03%, 35/66), followed by hypertension (40.91%, 27/66), agranulocytosis (16.67%, 11/66), chronic digestive system disease (11.4%, 16/140), and diabetes (15.15%, 10/66). Some patients are complicated with various comorbidities. The median length of hospital stay was 28.5 (20–55) days. Additionally, 18 (27.27%) patients required intensive care unit (ICU) admission, with a median ICU stay of 3.5 (1-7) days (**Table 1**). The overall mortality rate was 15.15% (10/66). Among the 10 deceased patients, seven had fungal bloodstream infections, while the remaining 3 deaths were attributed to *Acinetobacter baumannii* infection with myocardial infarction, *Pseudomonas aeruginosa* bloodstream infection, and COVID-19 associated septic shock, respectively (**Table 2**). Patient characteristics of the 10 deceased patients are detailed in **Table 2**. 16 patients presented with fever before treatment, and 6 patients remained fever after treatment (**Table 1**). Overall, specimen types comprised drainage fluid [n=23 (30.67%)], followed by BALF [n =23 (30.67%)], organ preservation cultures [n =15 (20.00%)], blood [n=13 (17.33%)], and sputum [n = 1 (1.33%)] (**Figure 1**).

**Table 1:** Baseline characteristics of the study cohort (n=66).

Characteristics	Cases (n =66)	
Demographics		
Male, n (%)	39 (59.1)	
Age (years), mean ± SD	40.7 ± 9.6	
Comorbidities, n (%)		
Hypertension	27 (40.9)	
Diabetes	10 (15.2)	
Anemia	35 (53.0)	
Agranulocytosis	11 (16.7)	
Clinical outcomes		
Hospital LOS (days), median (IQR)	28.5 (20, 55)	
ICU admission rate, n (%)	18 (27.3)	
ICU LOS (days), median (IQR)	3.5 (1, 7)	
Mortality, n (%)	10 (15.2)	
**Body temperature (n)**	**Before**	**After**
Normal X	50	60
≥37.3°C	16	6

ICU, intensive care unit; LOS, length of stay; IQR, interquartile range.

**Table 2:** Characteristics of died patients. .

No.	Sex	Age (years)	Hospital LOS (days)	ICU LOS (days)	Cause of death	Source of fungal	Pathogen
1	Male	29	17	0	*Acinetobacter baumannii* infection, Myocardial infarction	Drainage fluid	*Candida glabrata*
2	Female	47	67	10	COVID-19, Septic shock	Blood, Drainage fluid, Organ preservation cultures	*Rhizomucor pusillus*
3	Male	33	13	6	Septic shock	Blood	*Rhizomucor pusillus, Aspergillus flavus*
4	Male	44	25	0	Septic shock	Blood	*Lichtheimia ramasa*
5	Male	46	22	2	Cerebral infarction, Septic shock	BALF	*Aspergillus flavus, Lichtheimia corymbifera, Rhizomucor pusillus*
						Blood	*Lichtheimia corymbifera*
6	Female	46	86	4	COVID-19, *Pseudomonas aeruginosa* infection	Organ preservation cultures	*Aspergillus niger*
7	Female	24	21	15	COVID-19, Septic shock	Blood	*Pneumocystis jirovecii*
8	Female	63	9	7	COVID-19, Septic shock	Blood	*Pneumocystis jirovecii*
9	Female	53	80	7	Septic shock, COVID-19	Blood	*Candida parapsilosis*
10	Female	43	121	0	Septic shock, COVID-19	BALF	*Candida parapsilosis*

LOS, length of stay; ICU, intensive care unit; BALF, bronchoalveolar-lavage fluid.

### Distribution of fungal species detected by mNGS and CMTs

**Figure 2:**
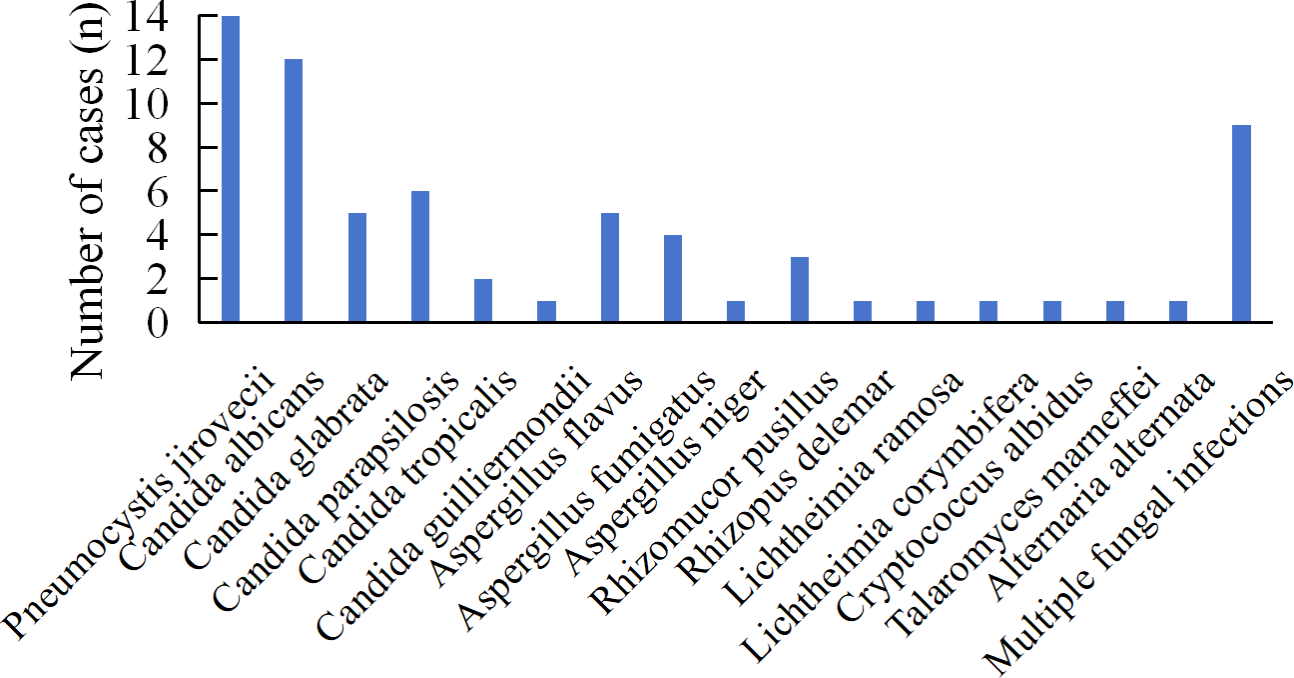
Distribution of fungi detected by mNGS; mNGS, metagenomic next-generation sequencing.

**Figure 3:**
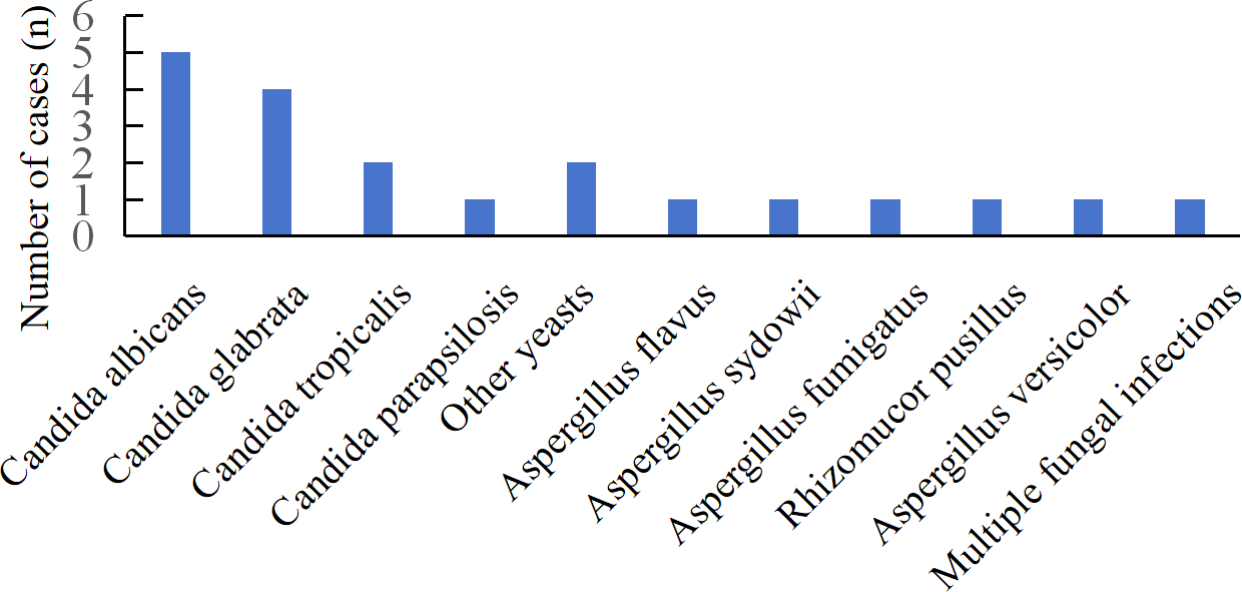
Distribution of fungi detected by CMTs; CMTs, conventional microbiological tests.

A total of 21 fungal species were identified among the 66 patients, with mNGS detecting 17 species (**Figure 2**). The most frequently detected pathogens by mNGS were *Pneumocystis jirovecii* (14)*, Candida albicans* (12), and *Candida parapsilosis* (6). mNGS identified 26 cases of *Candida* spp., 10 cases of *Aspergillus* spp., and 6 cases of *Mucor* spp. Nine patients were infected with multiple fungi, with specimens derived from BALF and blood in 7 cases and drainage fluid in 2 cases. Specific patient characteristics are presented in **Table 3**. CMTs yielded eleven species, with *Candida albicans* and *Candida glabrata* being most common. Only one patient demonstrated multiple fungi (**Figure 3**). CMTs identified 12 cases of *Candida* spp., 6 cases of *Aspergillus* spp., and 1 case of *Aspergillus* spp.

**Table 3:** Patients with multiple fungal pathogens.

Sex	Age (years)	Hospital LOS (days)	ICU LOS(days)	Outcomes	Source of fungal	Pathogen
Male	31	30	0	Improved	Drainage fluid	*Candida glabrata, Candida tropicalis*
Female	34	186	0	Improved	BALF	*Rhizomucor pusillus, Aspergillus fumigatus*
Male	33	13	6	Died	Blood	*Rhizomucor pusillus, Aspergillus flavus*
Male	38	22	0	Improved	Blood	*Candida parapsilosis, Candida tropicalis*
Female	44	192	0	Improved	BALF	*Aspergillus fumigatus, Aspergillus flavus, Candida albicans*
Male	28	29	0	Improved	BALF	*Aspergillus flavus, Aspergillus fumigatus, Rhizopus oryzae, Rhizopusdelemar, Pneumocystis jirovecii*
Male	46	22	2	Died	BALF	*Aspergillus flavus, Lichtheimia corymbifera, Rhizomucor pusillus*
Male	42	109	0	Improved	BALF	*Aspergillus flavus, Aspergillus terreus, Aspergillus niger*
Male	28	25	0	Improved	Drainage fluid	*Candida glabrata, Candida albicans*

LOS, length of stay; BALF, bronchoalveolar-lavage fluid.

**Figure 4:**
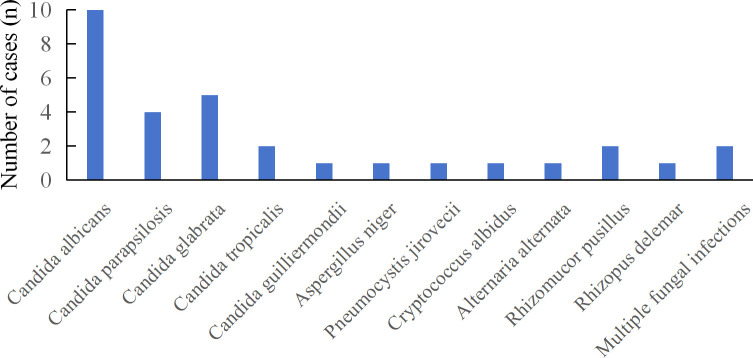
Distribution of fungi detected in drainage fluid and organ preservation fluids by mNGS.

**Figure 5:**
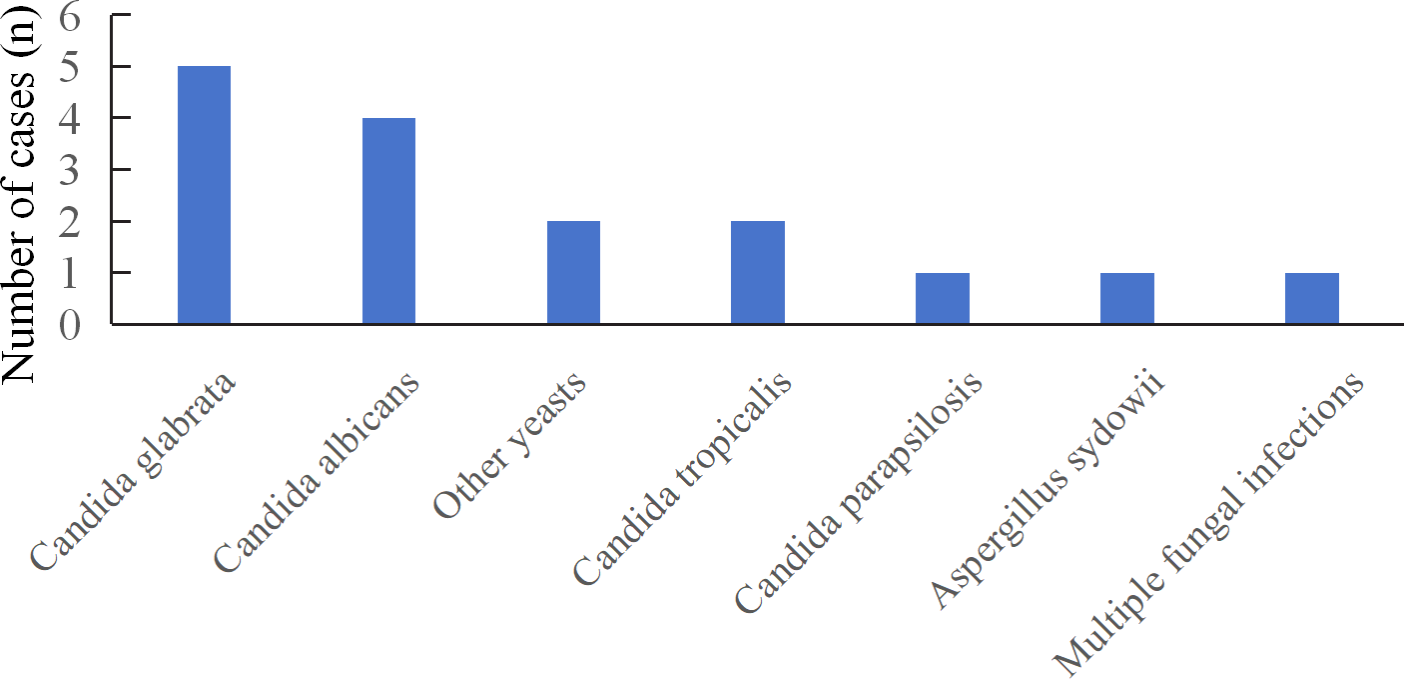
Distribution of fungi detected in drainage fluid and organ preservation fluids by CMTs.

DDI contributes to significant morbidity and mortality. Detection of pathogens in organ preservation fluid and drainage fluids may indicate donor-derived infection and facilitate risk mitigation. Consequently, thirty-eight samples from organ preservation and drainage fluids underwent further analysis. NGS identified 12 distinct fungal species from 31 fungal strains, with *Candida* spp., representing 70.97% (22/31) of all strains. Two patients were infected with multiple fungal species (**Figure 4**). Traditional cultures yielded sixteen cases of fungus, with *Candida* spp. accounting for 81.25% (13/16). Only one patient was infected with multiple fungi (**Figure 5**).

### Mixed infections detected by mNGS

Mixed infection was defined as the detection of two or more infectious pathogens. The mNGS method identified mixed infections in 57 samples. The most prevalent pattern was bacterial-fungal co-infection (21/75, 28%), followed by bacterial-viral-fungal triple infection (19/75, 25.33%). Fungal-viral co-infection was detected in 14 samples (14/75, 18.67%); eleven patients were diagnosed with fungal infection (11/75, 14.67%); three additional patients exhibited co-infections with *Mycobacterium tuberculosis* or atypical pathogens.

Notably, twenty-seven mixed infections were detected in organ preservation cultures and drainage fluid. The predominant pattern was bacterial-fungal co-infection (17/38, 44.74%), followed by bacterial-viral-fungal triple infection (8/38, 21.05%). Nine samples were identified with two or more fungal species, among which 3 patients were infected with two kinds of *Candida* spp., 4 with both *Aspergillus* spp., *Mucor* spp., 1 with multiple *Aspergillus* spp. and 1 with *Aspergillus* spp. and *Candida* spp. The 9 cases were derived from 5 alveolar lavage fluid, 2 cases from blood, and 2 cases from drainage fluid. Among them, two cases resulted in death: one from combined *Mucormycosis* and aspergillosis, and another from multiple *Mucormycosis* infections (**Table 3**).

### Comparison of the diagnostic performance of mNGS and CMTs

**Figure 6:**
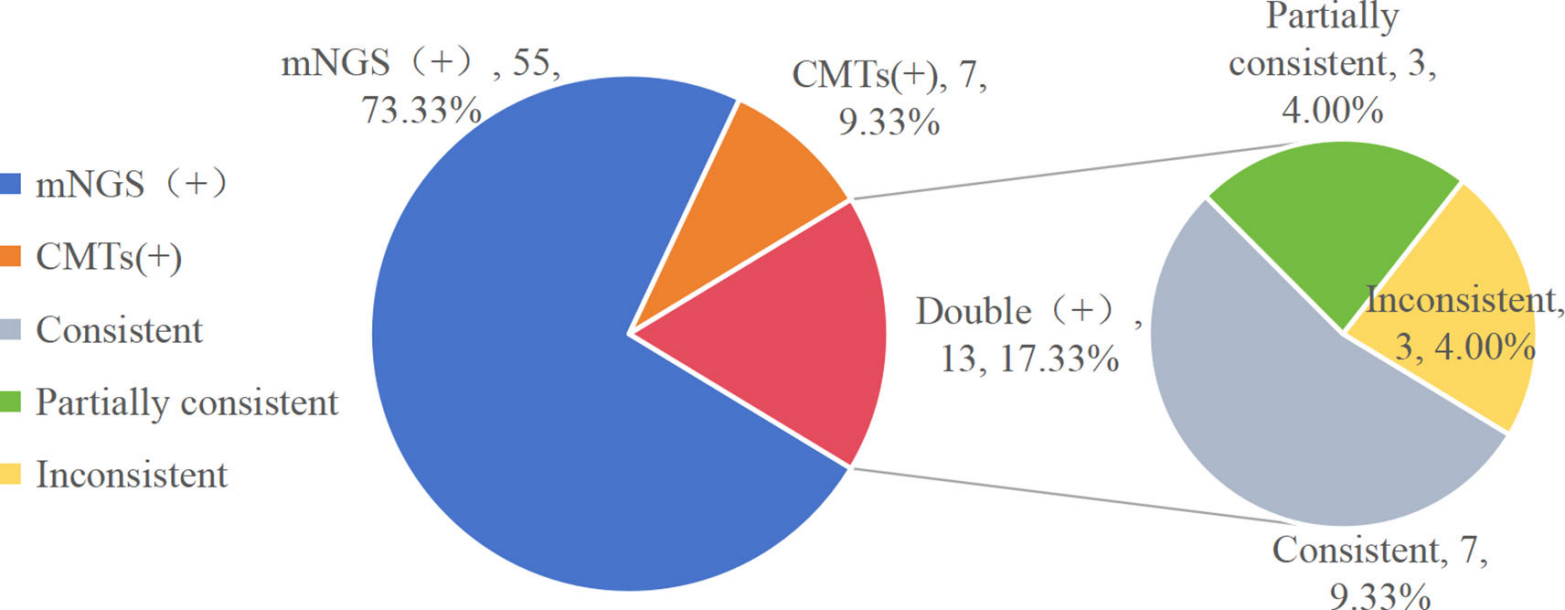
Consistency of mNGS and CMTs in fungal infections.

All samples underwent both mNGS and CMTs. In this study, mNGS yielded positive results for fungi in 68 of 75 (90.67%) patients, demonstrating marked superiority over CMTs at 26.67% (20/75). A comparison of diagnostic results between mNGS and CMTs is shown in **Figures 2**, **3**. In our study, mNGS and CMTs were both positive in 13 (13/75, 13.33%) cases. A total of 55 (55/75, 73.33%) cases were positive by mNGS only, whereas 7 (7/75, 9.33%) cases were positive by CMTs only. Among the 13 double-positive cases, concordance between mNGS and CMTs was complete in 7 (7/75, 9.33%), partial in 3 (3/75, 4%), and absent in 3 (3/75, 4%) (**Figure 6**).

Overall, mNGS identified more *candida* (26 vs 12), *Aspergillus* (10 vs 4), and *Mucor* (6 vs 2) organisms compared with CMTs. Pathogens missed by conventional culture included *Pneumocystis jirovecii* (14), *Candida guilliermondii* (1), *Aspergillus niger* (1), *Rhizopus delemar* (1), *Lichtheimia ramasa* (1), *Lichtheimia corymbifera* (1), *Cryptococcus albidus* (1), *Talaromyces marneffei* (1), A*lternaria alternata* (1), *Rhizopus oryzae* (1), and *Aspergillus terreus* (1). Conversely, pathogens missed by mNGS included yeast (1), *Aspergillus sydowii* (1), and *Aspergillus versicolor* (1). In summary, mNGS identified pathogens that were relatively complex or undetectable under standard culture conditions.

### Donor-derived fungal infections

From September 2021 to August 2023, 146 patients underwent kidney transplantation from donors after cardiac death. Organ preservation fluid and/or drainage fluid from all patients underwent mNGS and CMTs. A total of 248 samples of organ preservation fluid and drainage fluid were collected, with fungi detected in 41 samples from 35 patients. Donor-derived fungi were identified in 11 (11/146, 7.5%) patients, including 10 cases of *Candida* spp. and 1 case of *Mucor* spp. Among these 11 patients, two fatalities occurred: one from bacterial infection and the other from disseminated *Mucormycosis*. No deaths attributable to *Candida* infection were observed. Detection methods included reliance on mNGS in 4 cases, CMTs in 4 cases, and both mNGS and CMTs in the remaining 3 cases (**Table 4**).

**Table 4:** Characteristics of donor-derived fungal infections.

NO.	Sex	Age (years)	Hospital LOS (days)	Outcomes	Pathogen	Detection method
1	Male	29	17	Died	*Candida glabrata*	mNGS, CMTs
2	Male	31	30	Improved	*Candida glabrata*	mNGS, CMTs
3	Female	37	24	Improved	*Candida tropicalis*	CMTs
4	Male	38	51	Improved	*Candida glabrata*	mNGS, CMTs
5	Male	38	30	Improved	*Candida glabrata*	CMTs
6	Female	47	67	Died	*Rhizomucor pusillus*	mNGS
7	Female	35	57	Improved	*Candida albicans*	mNGS
8	Female	44	85	Improved	*Candida albicans*	mNGS
9	Male	39	27	Improved	*Candida albicans*	mNGS
10	Male	28	25	Improved	*Candida glabrata*	CMTs
11	Female	55	21	Improved	*Candida albicans*	CMTs

LOS, length of stay; CMTs, conventional microbiological tests; mNGS, metagenomic next-generation sequencing.

### Impacts of mNGS on the application of antibiotic treatment

We assessed the impact of mNGS on antimicrobial therapy, as detailed in **Table 5**, to determine its role in optimizing treatment decisions. The key finding was that mNGS results informed therapeutic adjustments in 42.4% (28/66) of patients. These adjustments manifested in two critical ways: firstly, by enabling targeted therapy, such as adding voriconazole for *Aspergillus* (n=10) or sulfamethoxazole for *Pneumocystis jirovecii* (n=10); and secondly, by facilitating therapy de-escalation, as evidenced by the discontinuation of caspofungin in 13 patients. For the majority of patients whose regimen remained unchanged, mNGS validated the adequacy of the initial empirical caspofungin therapy.

**Table 5:** Adjustment of antifungal therapy based on mNGS results.

Antifungal agent	Pre-mNGS (n)	Post-mNGS adjustment (n)	
		Reduced	Increased
Voriconazole	6	3	10
Posaconazole	0	0	6
Isavuconazole	0	0	1
Caspofungin	51	13	5
Amphotericin B	0	0	2
TMP-SMZ (therapeutic dose)	5	2	10
No change	38	–	–

mNGS, metagenomic next-generation sequencing; TMP-SMX, trimethoprim-sulfamethoxazole.

## Discussion

Effective management of post-transplant infections depends on prevention, early diagnosis, and targeted therapy. Although less frequent than bacterial infections, invasive fungal infections (IFIs) in transplant recipients are associated with high mortality rates, ranging from 15% to 50% in kidney transplant patients (Seok et al., 2020; van Delden et al., 2020; Wu et al., 2023). While mNGS has advanced infectious disease diagnostics, its specific application in profiling fungal infections in kidney transplant recipients remains underexplored. In this study, we conducted a comprehensive analysis of fungal infections in these patients using mNGS and compared its performance with CMTs. Our aim was to characterize the fungal pathogen spectrum, including mixed and donor-derived infections, and to evaluate the clinical impact of mNGS on antifungal therapy.

Currently, fungal smear and culture, serum (1,3)-b-D-glucan (G) or galactomannan (GM) tests, and PCR are used for microbiological fungal analysis. Traditional culture methods offer valuable information on drug susceptibility but have limitations such as low positive rates, reduced sensitivity, high false-positive rates, and lengthy processing times. Kidney transplant recipients often receive multiple medications, which may affect the accuracy of these diagnostic methods. Conventional blood cultures may fail to diagnose candidiasis in up to 25–50% of cases (Bassetti et al., 2016). In contrast, mNGS, characterized by rapid detection, high sensitivity, and broad coverage, effectively compensates for these shortcomings.

The significantly higher fungal detection rate by mNGS (90.67% vs. 26.67%) is consistent with emerging evidence from other immunocompromised cohorts (Decker et al., 2019; Chen et al., 2020; Chien et al., 2022; Wang et al., 2022; Overbeek et al., 2024). This discrepancy is especially notable for fastidious organisms like *Pneumocystis jirovecii*, which cannot be routinely cultured, and fungi with fragile structures prone to damage during transport or processing. Although most reports emphasize its advantages in fungal diagnosis, some studies note limitations, particularly for filamentous molds such as *Aspergillus* (Miao et al., 2018). One study even suggested that conventional methods may outperform mNGS in diagnosing pulmonary fungal infections (Peng et al., 2021), indicating that mNGS’s diagnostic value in mycology is still somewhat contested. However, the heightened sensitivity of mNGS introduces the critical challenge of distinguishing true invasive infections from colonization or environmental contamination, especially for organisms like *Candida* and *Aspergillus*, which can be commensal. In our practice, a positive mNGS result was never interpreted in isolation; clinical decisions were always based on integrating mNGS findings with the patient’s clinical trajectory, radiological findings, and immune status. This multi-parameter approach is essential to mitigate the risk of overdiagnosis and unnecessary treatment, converting a potentially high false-positive rate into clinically actionable insights.

A particular strength of mNGS is its ability to identify fastidious, non-culturable fungi, which is crucial in immunocompromised hosts vulnerable to opportunistic pathogens. Consistent with other studies reporting frequent detection of *Pneumocystis jirovecii*, *Candida*, and *Aspergillus* (Zhao et al., 2021; Wang et al., 2022; Fishman, 2017), our findings confirm the utility of mNGS in profiling the fungal spectrum in kidney transplant recipients. Notably, mNGS demonstrates exceptional performance in diagnosing *Pneumocystis jirovecii*, a major pathogen in this population, with sensitivity and specificity surpassing conventional staining and biomarker assays (Jiang et al., 2021; Xu et al., 2021; Duan et al., 2022; Sun et al., 2022). Regarding specific pathogens, *Candida* species remain the most prevalent fungi among solid organ transplant recipients, a pattern also evident in our cohort. Invasive aspergillosis continues to carry a poor prognosis, especially in lung transplant recipients (Singh and Husain, 2013). Increasingly, studies - including ours - support mNGS for superior detection of *Aspergillus* compared to traditional methods (Hoenigl et al., 2023; Shi et al., 2023; Zhan et al., 2023; Niu et al., 2024), underscoring its emerging role in managing these high-risk infections.

mNGS demonstrates strong performance in identifying fungal species and co-infecting pathogens, highlighting its potential to guide antimicrobial therapy. Multiple studies have confirmed the clear advantages of mNGS in detecting mixed infections (Liang et al., 2022; Zhang et al., 2022), consistent with our findings. Additionally, mNGS identified polymicrobial fungal infections in 9 samples, whereas CMTs detected only one. The comprehensive fungal profile provided by mNGS offers valuable epidemiological insights into fungal infections among transplant recipients.

The impact of antibacterial drugs on mNGS is less pronounced than on CMTs, allowing tailored treatment strategies based on these findings. Accurate strain identification is crucial for guiding antifungal treatment. Several studies reported that mNGS prompted treatment modifications in 39.3%–45.1% of cases (Liang et al., 2022; Zhang et al., 2022; Shi et al., 2023). Consistent with these reports, our findings confirm that mNGS directly influences antifungal management by supporting informed initiation of targeted therapy and guided discontinuation of unnecessary medication. The rapid availability of mNGS results delivers timely microbiological evidence to support clinical decision-making, facilitating antimicrobial stewardship and potentially improving patient outcomes.

A key clinical finding of our study pertains to DDIs. Although less common than bacterial or viral counterparts, DDIs pose a severe threat in transplantation, with an overall DDI-associated mortality of 15% (Kaul et al., 2021). Current guidelines emphasize blood and urine cultures from deceased donors but do not routinely recommend culturing preservation fluid (Singh et al., 2012; Malinis and Boucher, 2019). A considerably higher DDI rate of 7.5% was identified in our cohort, where both mNGS and CMTs were systematically applied to preservation and drainage fluids. This contrasts with the 4% incidence reported in an earlier meta-analysis (Oriol et al., 2018). The elevated donor-derived fungal infection rate in our study may be attributed to the combined detection strategy employing both mNGS and CMTs for detection. A study further note that renal transplant recipients are at elevated risk for urinary tract infections and systemic complications from donor-derived pathogens, highlighting the importance of broad-spectrum screening strategies like those employed in our study (Fiorentino et al., 2019). Prior research indicates that donor-derived fungal infections are predominantly caused by *Candida* (24%), *Cryptococcus* (20%), and *Aspergillus* (13%), with associated mortality rates of 10.0%, 7.7%, and 33.3%, respectively (Kaul et al., 2021). In our study, all donor-derived *Candida* infections were successfully managed without recipient mortality, which may reflect our center’s protocol of routine postoperative caspofungin prophylaxis and subsequent therapy adjustments informed by etiological evidence. This outcome under*mucor*scores the importance of targeted antifungal strategies in recipients of grafts from donors with fungal colonization, consistent with current guideline recommendations (Singh et al., 2012). One fatal case of donor-derived *mycosis* was also identified, underscoring the indispensable role of sensitive detection tools like mNGS in identifying rare but high-risk pathogens that conventional screening might fail to detect.

The present study had several limitations. First, traditional detection methods such as the G test, GM test, cryptococcal capsular polysaccharide antigen test, and Grocott methenamine silver stain were not comprehensively employed. Fungi like *Mucor* and *Pneumocystis jirovecii* are difficult to culture, and these detection methods help diagnose these fungi. Therefore, a direct comparison of mNGS with these methods was not feasible. Second, mNGS cannot reliably distinguish between colonization and active infection, as fungi like *Candida* or *Aspergillus* commonly exist as commensals. Therefore, positive results require cautious interpretation within the full clinical context-including symptoms, radiological findings, and immune status-to avoid overdiagnosis and unnecessary treatment. Third, mNGS does not provide susceptibility results, although fungal resistance mechanisms tend to be less complex than bacterial resistance. Fourth, the thick cell wall of *Aspergillus* complicates nucleic acid extraction, leading to a false-negative result.

In summary, while mNGS enables rapid and sensitive detection of fungal infections-particularly those caused by unculturable pathogens-in kidney transplant recipients, its clinical application necessitates standardized yet adaptable protocols tailored to individual patient scenarios.

## Data Availability

The original contributions presented in the study are included in the article/supplementary material. Further inquiries can be directed to the corresponding author.
